# Role of Striatal-Enriched Tyrosine Phosphatase in Neuronal Function

**DOI:** 10.1155/2016/8136925

**Published:** 2016-04-12

**Authors:** Marija Kamceva, Jessie Benedict, Angus C. Nairn, Paul J. Lombroso

**Affiliations:** ^1^Child Study Center, Yale University School of Medicine, New Haven, CT 06519, USA; ^2^Department of Psychiatry, Yale University School of Medicine, New Haven, CT 06519, USA

## Abstract

Striatal-enriched protein tyrosine phosphatase (STEP) is a CNS-enriched protein implicated in multiple neurologic and neuropsychiatric disorders. STEP regulates key signaling proteins required for synaptic strengthening as well as NMDA and AMPA receptor trafficking. Both high and low levels of STEP disrupt synaptic function and contribute to learning and behavioral deficits. High levels of STEP are present in human postmortem samples and animal models of Alzheimer's disease, Parkinson's disease, and schizophrenia and in animal models of fragile X syndrome. Low levels of STEP activity are present in additional disorders that include ischemia, Huntington's chorea, alcohol abuse, and stress disorders. Thus the current model of STEP is that optimal levels are required for optimal synaptic function. Here we focus on the role of STEP in Alzheimer's disease and the mechanisms by which STEP activity is increased in this illness. Both genetic lowering of STEP levels and pharmacological inhibition of STEP activity in mouse models of Alzheimer's disease reverse the biochemical and cognitive abnormalities that are present. These findings suggest that STEP is an important point for modulation of proteins required for synaptic plasticity.

## 1. Introduction

There are 107 protein tyrosine phosphatases (PTPs) in the human genome and many of these play important roles in cellular function [1]. Striatal-enriched protein tyrosine phosphatase (STEP), encoded by the* PTPN5* gene, is a CNS-enriched member of the PTP family [2]. PTPs are divided into tyrosine-specific phosphatases and dual-specificity phosphatases, with tyrosine-specific phosphatases further divided into intracellular PTPs and receptor-like PTPs [3]. STEP is an intracellular PTP, expressed throughout the CNS with the exception of the cerebellum [4].

Dysfunction in a growing number of PTPs contributes to the etiology of several diseases [5–7] and, as a result, PTPs, including STEP, have emerged as attractive targets for drug discovery [8, 9]. The current model of STEP function is that it normally opposes synaptic strengthening by dephosphorylating key synaptic substrates. Substrates include subunits of glutamate receptors N-methyl-D-aspartate receptors (NMDARs) and *α*-amino-3-hydroxy-5-methyl-4-isoxazolepropionic acid receptors (AMPARs), leading to internalization of these receptor complexes [10–13]. Thus, increased expression of STEP disrupts synaptic function and is associated with a number of neuropsychiatric disorders, such as Alzheimer's disease [14–17]. Pharmacological inhibition of STEP would be predicted to alleviate synaptic dysfunction in Alzheimer's disease, and the successful effort in this area is reviewed below.

## 2. Domain Structure of Major STEP Isoforms

Like other PTPs, STEP contains a signature consensus sequence [I/V]HCxAGxxR[S/T]G at its C-terminus that is required for catalytic function and an upstream kinase-interacting motif (KIM) that is involved in binding to all known substrates [18–22]. The STEP family is alternatively spliced from a single STEP gene (*PTPN5*) and has two major isoforms, STEP_61_ and STEP_46_, which are differentially expressed in brain regions and at developmental times [18, 23, 24]. STEP_61_ is found in multiple brain regions that include the striatum, central nucleus of the amygdala, optic nerve, hippocampus, neocortex, spinal cord, olfactory tubercle and bulb, and lateral amygdala, while STEP_46_ is expressed in striatum, nucleus accumbens, amygdala, and the optic nerve [23, 25]. STEP_61_ is abundantly expressed at birth and throughout adulthood, while STEP_46_ is not expressed until postnatal day 6 and increases over the first postnatal month when it plateaus to adult levels [24, 26]. STEP isoforms are found in both excitatory and inhibitory neurons [27], as well as in glia [25, 28].

STEP_61_ contains 172 additional amino acids at its amino-terminus compared to STEP_46_. The region contains two hydrophobic domains that are required to target STEP_61_ to the endoplasmic reticulum and the postsynaptic density of dendritic spines [23, 29]; in contrast, STEP_46_ is primarily cytosolic [18]. STEP_61_ has two polyproline-rich regions that, in addition to the KIM domain, are involved in substrate binding and contribute to substrate specificity: the first polyproline domain is necessary for binding to Fyn [30], while the second is necessary for binding of Pyk2 [21].

Two additional alternatively spliced isoforms of STEP exist: STEP_38_ and STEP_20_ [4, 18, 31, 32]. While STEP_61_ and STEP_46_ both contain the signature consensus PTP sequence, STEP_38_ and STEP_20_ do not and are catalytically inactive [31]. Although these STEP isoforms remain to be fully characterized, they both contain KIM domains, suggesting that they may serve as variants that associate with target substrates and protect them from dephosphorylation. Both of these inactive STEP isoforms contain a 10-amino acid sequence at their carboxyl termini that is introduced during splicing and serves an unknown function.

## 3. Posttranslational Regulation of STEP

It is important to briefly review the posttranslational regulation of STEP as it informs us of potential mechanisms in disease. STEP activity is regulated by several mechanisms that include phosphorylation, dimerization, proteolytic cleavage, ubiquitination, and local translation (for more extensive review, see [33]). Two of these mechanisms of normal STEP regulation, phosphorylation and ubiquitination, are important to note when understanding STEP dysregulation in Alzheimer's disease, which is discussed below.

Phosphorylation by protein kinase A (PKA) reduces STEP activity in two ways. PKA directly phosphorylates STEP_61_ and STEP_46_ at a regulatory serine within their KIM domains [34], introducing steric hindrance that prevents STEP from binding to its substrates. PKA also reduces STEP activity indirectly by phosphorylating DARPP-32, a potent inhibitor of protein phosphatase 1 (PP1). PP1 normally dephosphorylates STEP at the regulatory serine residue within the KIM domain; thus, inhibition of PP1 maintains STEP phosphorylation and reduces levels of the dephosphorylated, active STEP protein [35].

## 4. STEP Substrates

### 4.1. Mitogen-Activated Protein Kinase (MAPK) Family

The discovery of STEP substrates was an important advance in the understanding of the possible function of STEP in regulating neuronal signaling. Two members of the MAPK family of proteins are STEP substrates, the extracellular signal-regulated kinases 1 and 2 (ERK1/2) and p38 [36–39]. ERK1/2 is implicated in synaptic plasticity and memory formation via its roles in stabilizing dendritic spines, initiating local protein synthesis in dendrites and spines, and involvement in nuclear transcription [40, 41]. STEP dephosphorylates the regulatory Tyr^204^ or Tyr^187^ residues in their respective activation loops, thereby inactivating ERK1/2.

The role of STEP regulation of ERK1/2 signaling has been studied in numerous ways, including infusion of a membrane-permeable TAT- (transactivator of transcription-) STEP cysteine to serine mutant [TAT-STEP (C to S)]. This mutant isoform is catalytically inactive, as the cysteine residue is required for substrate dephosphorylation. However, TAT-STEP (C to S) still binds to its substrates but does not release them, as dephosphorylation is required for substrate release; thus, TAT-STEP (C to S) inhibits downstream signaling pathways [10, 37]. ERK1/2 is necessary for the development of synaptic strengthening and the consolidation of fear memories in the lateral amygdala (LA). Infusion of TAT-STEP (C to S) into the LA rats did not affect the acquisition of fear memories, but there was no consolidation of these memories [42]. STEP knockout (KO) mice further established a relationship between STEP and ERK1/2, as these mice have significant elevation of phospho-ERK1/2 and increased phosphorylation of the downstream targets of ERK1/2, the transcription factors CREB and Elk1 [43, 44]. Moreover, STEP KO mice have facilitated amygdala-dependent learning (fear conditioning [45]) and facilitated hippocampal-dependent learning (Morris water maze [44]). These studies suggested that STEP normally regulates the duration of ERK1/2 signaling and also suggested the hypothesis that elevated levels of STEP might disrupt synaptic plasticity and memory formation [37].

The MAPK, p38, is also a STEP substrate but in contrast to ERK1/2 is involved in regulation of cell death pathways and NMDAR-mediated excitotoxicity [46, 47]. Excess glutamate stimulation activates extrasynaptic GluN2B-containing NMDARs, which results in phosphorylation of p38; p38 then phosphorylates target proteins involved in cell death pathways [48]. STEP normally dephosphorylates Tyr^182^ in the activation loop of p38, inactivating the protein [13, 48]. In addition, a number of studies have used molecular, kinetic, and structural analyses to gain insights into small differences in the KIM-containing PTPs that affect their binding to ERK2 and p38 [49–52]. Notably, both ERK1/2 and p38 regulate STEP expression levels through modulation of two phosphorylation sites adjacent to the KIM domain and dephosphorylation of these sites leads to the ubiquitination and degradation of STEP, suggesting a feedback mechanism to decrease STEP expression when ERK1/2 and p38 levels are low [53].

A study by Xu and colleagues [48] shed light on how STEP might regulate both p38 and ERK1/2, two proteins with very different and opposing functions. The differential regulation of these kinases by STEP depended on whether synaptic or extrasynaptic NMDARs were stimulated. STEP_61_ is rapidly ubiquitinated and degraded following synaptic NMDAR stimulation, resulting in activation of ERK1/2 (but not p38 signaling) and activation of synaptic strengthening and neuronal survival pathways. With increased glutamate signaling, extrasynaptic NMDARs are engaged and promote activation of calpain and the cleavage of STEP_61_ within the KIM domain. The cleavage of the substrate-binding domain results in a STEP variant (STEP_33_) that is unable to bind to and inactivate its substrates. Thus, stimulation of extrasynaptic NMDARs results in cleavage of STEP_61_ and activation of p38 and cell death pathways. Using a peptide corresponding to the calpain cleavage site that prevents STEP_61_ cleavage, there was a significant protection of neurons from glutamate-mediated excitotoxicity [48].

### 4.2. GluN2B and GluA2

Early studies demonstrated that dopamine signaling regulates STEP activity [34]. As mentioned above, stimulation of dopamine D1 receptors leads to activation of PKA and the phosphorylation and inactivation of STEP. Stimulation of D2 receptors has the opposite effects by reducing phosphorylation of the regulatory serine residue within the KIM domain and promoting the dephosphorylation of STEP substrates [34]. Thus, the hypothesis emerged that perhaps STEP lay between dopamine signaling and glutamate signaling through the ability of dopamine to regulate STEP activity and thereby regulate the tyrosine phosphorylation and surface expression of both NMDA and AMPA receptor complexes [10, 12, 44, 54].

Glutamate is the most abundant excitatory neurotransmitter within the CNS and binds to both metabotropic and ionotropic glutamate receptors to promote numerous cell signaling pathways in neurons [55, 56]. NMDARs are ligand-gated ion channels composed of two GluN1 and two GluN2 subunits. Activation of NMDARs requires both glutamate and glycine binding to the receptor as well as postsynaptic membrane depolarization. These receptors are selectively permeable to Ca^2+^ ions, which activate signaling molecules needed for long-term potentiation (LTP) and long-term depression (LTD) [57, 58]. STEP regulates the phosphorylation of the GluN2B subunit of NMDARs via two parallel pathways, the direct dephosphorylation of GluN2B (Tyr^1472^) as well as inactivation of the nonreceptor tyrosine kinase Fyn that phosphorylates GluN2B at that site [30, 59]. When dephosphorylated by STEP, the Tyr^1472^ residue of GluN2B binds to clathrin adaptor proteins and promotes internalization of GluN1/GluN2B receptors [60]. Congruent with this observation, the surface expression of GluN1/GluN2B receptor complexes is increased in STEP KO mice [14, 44].

The effect of STEP on NMDAR function is significant. High levels of STEP decrease NMDAR excitatory postsynaptic currents (EPSCs) and prevent the occurrence of high-frequency stimulation LTP [54]. When STEP was inhibited with a functional-inhibiting STEP antibody, NMDAR EPSCs were enhanced and LTP occluded. The administration of a noncompetitive NMDAR agonist dizocilpine (MK801) and a Src family kinase inhibitory peptide prevents these effects, suggesting a role of STEP as a “tonic brake” on LTP by opposing Src family kinase-mediated enhancement of NMDARs activity [54].

As noted above, STEP is rapidly ubiquitinated and degraded after synaptic NMDAR stimulation [48], consistent with the emerging model that STEP activity must be decreased for LTP to occur. This is consistent with a recent study that found a role for STEP in the regulation of homeostatic synaptic plasticity [61]. Prolonged neuronal activity results in the upregulation of STEP that increases removal of NMDA and AMPA receptors from synaptic membranes. Prolonged neuronal inhibition had the opposite effect, leading to the hypothesis that fine-tuning of STEP activity is necessary for maintaining proper levels of these glutamate receptors at synapses.

AMPARs are also implicated in synaptic strengthening and memory consolidation. These receptors are ligand-gated ion channels composed of subunits GluA1 to GluA4. They regulate fast synaptic transmission that depolarizes postsynaptic membranes and activates NMDARs [56, 62]. AMPAR trafficking occurs in LTD and appears to be regulated by tyrosine phosphatases that include STEP [12, 63, 64]. STEP was found to regulate the Tyr dephosphorylation of the GluA2 subunit, leading to internalization of GluA1/GluA2 receptor complexes following mGluR stimulation [12].

Local translation of STEP is increased after activation of mGluRs by the agonist DHPG (S-3,5-dihydroxyphenylglycine). This results in the tyrosine dephosphorylation of the GluA2 subunit and internalization of GluA1/GluA2 receptor complexes [12]. DHPG induces the dephosphorylation of GluA2 and internalization of AMPARs, which is decreased by the substrate-trapping protein TAT-STEP (C to S). Further, STEP KO neuronal cultures do not undergo DHPG-mediated AMPAR endocytosis, which is restored with the addition of wild type TAT-STEP protein to the STEP KO cultures. These findings suggested that, following mGluR stimulation, STEP is activated to dephosphorylate GluA2 receptors, promoting their internalization. As suggested by this model, the surface expression of GluA1/GluA2-containing AMPARs is elevated in STEP KO mice [12, 44].

## 5. STEP Dysregulation in Alzheimer's Disease

The dysregulation of STEP and glutamate receptors is implicated in several neuropsychiatric disorders, including Alzheimer's disease (AD) [65, 66]. In AD, abnormally high levels of A*β* bind to and activate *α*7nAChRs [67–70], causing calcium influx and activation of calcineurin and PP1 and the dephosphorylation of STEP at the regulatory serine site within the KIM domain [10]. The ability of STEP to bind to its target proteins is increased and STEP substrates are dephosphorylated. To confirm that A*β* binding to *α*7nAChRs and activation of PP1 were leading to activation of STEP, neuronal cultures derived from *α*7nAChR KO mice and treated with A*β* were used to test whether activation of STEP was prevented in the absence of *α*7nAChRs. In fact, there was only a partial reduction STEP activation, suggesting that another mechanism was involved in activating STEP in AD.

Both mouse models of AD and neuronal cultures treated with A*β* were examined and found to have an accumulation of active STEP [14, 71–73]. The increase in STEP was shown not to be due to transcription or translation, suggesting that perhaps the normal degradation of STEP was disrupted. One of the effects of A*β* is inhibition of the proteasome [74, 75]. Since STEP is normally degraded through the ubiquitin proteasome pathway, an increase in STEP activity was found to be due to an A*β* disruption of the ubiquitin proteasome pathway. In summary, an increase in the dephosphorylation of STEP coupled with a decrease in its degradation leads to the significant increase in STEP activity in AD.

## 6. Studies of STEP in Mouse Models of AD

*Tg-2576.* The Tg-2576 AD model mouse line is a transgenic mouse line that overexpresses the 695-amino acid isoform of human amyloid precursor protein (APP). APP is an integral membrane protein, proteolysis of which generates the amyloid fibrillar form of A*β*, the primary component in amyloid plaques in AD brains. The mutated APP present in this mouse line contains Lys^670^  → Asn and Met^671^  → Leu mutations [76] and these mutations in APP are found in early onset familial AD [77–79]. At 3 months of age, Tg-2576 mice perform normally in cognitive tasks and A*β* levels are indistinguishable from control animals. However, the Tg-2576 mice show cognitive impairments by 10 months of age [76]. STEP levels are normal at the earlier time points but are significantly elevated at later time points [72].

*3xTg-AD.* The 3xTg-AD transgenic mouse line possesses three separate mutations. First, the 3xTg-AD line has the same APP mutation present in Tg-2576 mice. Second, 3xTg-AD mice have a presenilin mutation, one of the proteins comprising the *γ*-secretase complex responsible for cleaving APP at the C-terminus of the A*β* domain. Third, tau is mutated in the 3xTg-AD line. Tau is a microtubule-associated protein acting to stabilize microtubules by binding to tubulin. Tau is hyperphosphorylated in AD, which causes paired helical filaments and destabilization of microtubules. These paired helical filaments are found in neurofibrillary tangles in patients with AD [80].

The 3xTg-AD mouse line has several phenotypes consistent with symptoms of human AD [81, 82]. Working memory and hippocampal memory deficits are documented, as are circadian rhythm abnormalities that are often present in early stages of AD. These behavioral and cognitive deficits are seen in combination with A*β* plaque aggregation and neurofibrillary tangles, which include paired helical filaments of hyperphosphorylated tau protein [81, 82]. STEP levels were again found to be normal at earlier time points when cognitive function was unaffected but were significantly elevated at time points when cognitive deficits were present. Moreover, crossing 3xTg-AD mice with STEP KO mice reversed the cognitive deficits [14, 72].

## 7. STEP Inhibition and AD Mouse Models

The elevation of STEP in AD as well as the finding that genetic reduction of STEP reversed cognitive deficits in an AD mouse model validated STEP as a target for drug discovery. A high throughput screen led to the discovery of an inhibitor of STEP, 8-(trifluoromethyl)-1,2,3,4,5-benzopentathiepin-6-amine hydrochloride (TC-2153) [83]. Cortical neurons treated with TC-2153 exhibit significant increase in the Tyr phosphorylation of STEP substrates GluN2B, Pyk2, and ERK1/2. Mice injected with TC-2153 also showed increased Tyr phosphorylation of STEP substrates. Phosphatase assays were performed comparing inhibition of STEP to a panel of PTPs, including two highly related PTPs, He-PTP and PTP-SL. TC-2153 was more selective towards STEP compared with these other PTPs. Furthermore, STEP is only found in the cortex, whereas the highly related He-PTP is found in the spleen and PTP-SL in the cerebellum, tissues that lack STEP. WT and STEP KO mice were injected with TC-2153 or vehicle and the Tyr phosphorylation of ERK1/2 and Tyr phosphorylation of Pyk2 were compared in various organs. Significant increases in pERK1/2 and pPyk2 were observed only in the frontal cortex and hippocampus, but not in tissues outside of the brain or the cerebellum, where other members of the PTP family dephosphorylate ERK1/2 and Pyk2 but do not appear to be inhibited by TC-2153.

To determine the mechanism by which TC-2153 inhibits STEP, glutathione (GSH) was added in* in vitro* assays. It decreased the activity of TC-2153 by two orders of magnitude, implying an oxidative mechanism for STEP inhibition. STEP was then incubated with TC-2153 to monitor enzyme activity. Following 24 h of dialysis, STEP remained inhibited, suggesting that TC-2153 led to the formation of a covalent bond, although STEP activity could be recovered following incubation with GSH or DTT.

High-resolution tandem mass spectrometry was performed to determine whether TC-2153 modified the active site cysteine of STEP. WT STEP and a STEP mutant in which the catalytic cysteine was changed to serine were compared. Analysis of the catalytic Cys^472^ of STEP in the absence of TC-2153 revealed a disulfide bridge between Cys^465^ and Cys^472^ which was not present in the STEP (C to S) mutant. Incubation of WT STEP with TC-2153 revealed the presence of a* de novo* trisulfide within the Cys^465^/Cys^472^ bridge, which was not observed in WT STEP alone or in the mutated STEP. These results suggested that the active site cysteine is modified by TC-2153 and that sulfur(s) from the benzopentathiepin core is retained.

TC-2153 was effective in reversing cognitive deficits in both 6- and 12-month-old 3xTg-AD mice [83]. In the novel object recognition task (NOR), mice were injected with either vehicle or TC-2153 three hours prior to the training phase and tested for memory recall after 24 hours. Post hoc analysis suggested that discrimination indexes for object memory in the AD-TC group were significantly higher than those of the AD-Veh group, while TC-2153-treated WT mice did not differ from the Veh-treated WT mice. Of interest, no significant changes were found for beta amyloid or phospho-tau levels in 12-month-old 3xTg-AD mice, suggesting that inhibition of STEP activity was sufficient to reverse cognitive deficits.

The reference memory version of the Morris water maze was then conducted [83]. 3xTg-AD mice were injected daily with TC-2153, 3 hours prior to training for peak efficacy. This STEP inhibition resulted in a reversal of memory deficits on days 5 and 6 of the task in 3xTg-AD mice. To quantify memory status, the number of mouse entries in a circular zone located around the platform, or the target zone, and in the opposite quadrants was evaluated during probe trial 24 hours after the last acquisition day. AD mice showed no preference for the target zone, in contrast to AD mice treated with TC-2153, which spent as much time as WT mice in the target zone.

## 8. Conclusion

**Figure 1:**
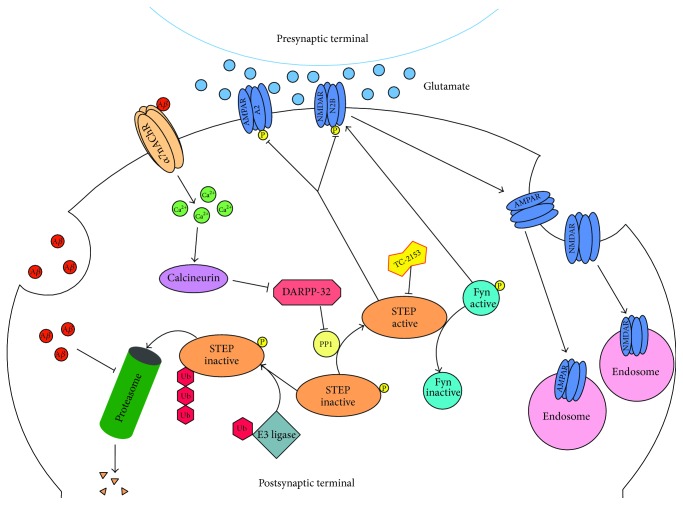
STEP signaling pathways associated with Alzheimer's disease. The binding of A*β* to *α*7nAChRs results in activation of calcineurin (PP2B), inhibition of DARPP-32, and activation of PP1. PP1 dephosphorylates STEP_61_ at a regulatory serine within the substrate-binding domain (Ser^221^). Dephosphorylation of this serine residue increases the affinity of STEP for its substrates. In a parallel pathway, A*β* inhibits the proteasome, thereby blocking the degradation of STEP_61_. Both mechanisms result in an accumulation of active STEP_61_. The increase in active STEP_61_ results in increased dephosphorylation of GluN2B Tyr^1472^ and internalization of GluN2B-containing NMDARs. In addition, dephosphorylation of Fyn results in its inactivation. Thus, active STEP_61_ directly dephosphorylates GluN2B and at the same time inactivates the kinase that phosphorylates STEP_61_ at Tyr^1472^.

STEP acts by dephosphorylating regulatory tyrosine residues in substrates that include subunits of both NMDA and AMPA glutamate receptors, thereby leading to internalization of these receptor complexes (see Figure 1). Additional targets of STEP include the kinases ERK1/2, Fyn, and Pyk2 that are inactivated by dephosphorylation of regulatory tyrosines within their activation loop, thus modulating downstream signaling pathways. When STEP activity is elevated, as occurs in Alzheimer's disease, the increased internalization of glutamate receptors disrupts synaptic function and contributes to the cognitive deficits that are present. Importantly, the STEP inhibitor TC-2153 significantly improves cognitive function in 3xTg-AD mice.

Although this review focused on Alzheimer's disease, STEP activity is elevated in several additional disorders, including Parkinson's disease [17], drug abuse [84–86], fragile X syndrome [16], and schizophrenia [15]. Moreover, a series of papers recently showed that low levels of STEP also disrupt synaptic function in several additional disorders, including Huntington's chorea [87, 88], cerebral ischemia [89], alcohol abuse [90–92], and stress disorders [93–95]. Thus the current model suggests that both high and low levels of STEP activity disrupt signaling pathways and contribute to neuropsychiatric and neurodegenerative disorders, making STEP an important focus of future research.
